# Alterations of the renin angiotensin system in human end-stage heart failure before and after mechanical cardiac unloading by LVAD support

**DOI:** 10.1007/s11010-020-03787-7

**Published:** 2020-06-20

**Authors:** Rebecca Messmann, Alexander Dietl, Stefan Wagner, Oliver Domenig, Carsten Jungbauer, Andreas Luchner, Lars S. Maier, Simon Schopka, Stephan Hirt, Christof Schmid, Christoph Birner

**Affiliations:** 1 Department of Internal Medicine II, University Hospital Regensburg, Regensburg, Germany; 2 Attoquant Diagnostics, Vienna, Austria; 3 Department of Cardiology, Clinic Barmherzige Brüder, Regensburg, Germany; 4 Department of Cardiothoracic Surgery, University Hospital Regensburg, Regensburg, Germany; 5 Department of Internal Medicine I, Klinikum St. Marien, Amberg, Germany

**Keywords:** Heart failure, LVAD, Renin angiotensin system

## Abstract

Heart transplantation is often an unrealizable therapeutic option for end-stage heart failure, which is why mechanical left ventricular assist devices (LVADs) become an increasingly important therapeutic alternative. Currently, there is a lack of information about molecular mechanisms which are influenced by LVADs, particularly regarding the pathophysiologically critical renin angiotensin system (RAS). We, therefore, determined regulation patterns of key components of the RAS and the β-arrestin signaling pathways in left ventricular (LV) tissue specimens from 8 patients with end-stage ischemic cardiomyopathy (ICM) and 12 patients with terminal dilated cardiomyopathy (DCM) before and after LVAD implantation and compared them with non-failing (NF) left ventricular tissue samples: AT1R, AT2R, ACE, ACE2, MasR, and ADAM17 were analyzed by polymerase chain reaction. ERK, phosphorylated ERK, p38, phosphorylated p38, JNK, phosphorylated JNK, GRK2, β-arrestin 2, PI3K, Akt, and phosphorylated Akt were determined by Western blot analysis. Angiotensin I and Angiotensin II were quantified by mass spectrometry. Patients were predominantly middle-aged (53 ± 10 years) men with severely impaired LV function (LVEF 19 ± 8%), when receiving LVAD therapy for a mean duration of 331 ± 317 days. Baseline characteristics did not differ significantly between ICM and DCM patients. By comparing failing with non-failing left ventricles, i.e., before LVAD implantation, a downregulation of AT1R, AT2R, and MasR and an upregulation of ACE, ACE2, GRK, β-arrestin, ERK, PI3K, and Akt were seen. Following LVAD support, then angiotensin I, ACE2, GRK, and β-arrestin were downregulated and AT2R, JNK, and p38 were upregulated. ACE, angiotensin II, AT1R, ADAM17, MasR, ERK, PI3K, and Akt remained unchanged. Some regulation patterns were influenced by the underlying etiology of heart failure, the severity of LV dysfunction at baseline, and the duration of LVAD therapy. Key components of the RAS and β-arrestin signaling pathways were divergently altered in failing left ventricles both before and after LVAD implantation, whereas a remarkable fraction remained unchanged. This indicates a rather incomplete molecular reverse remodeling, whose functional relevance has to be further evaluated.

## Introduction

**Fig. 1:**
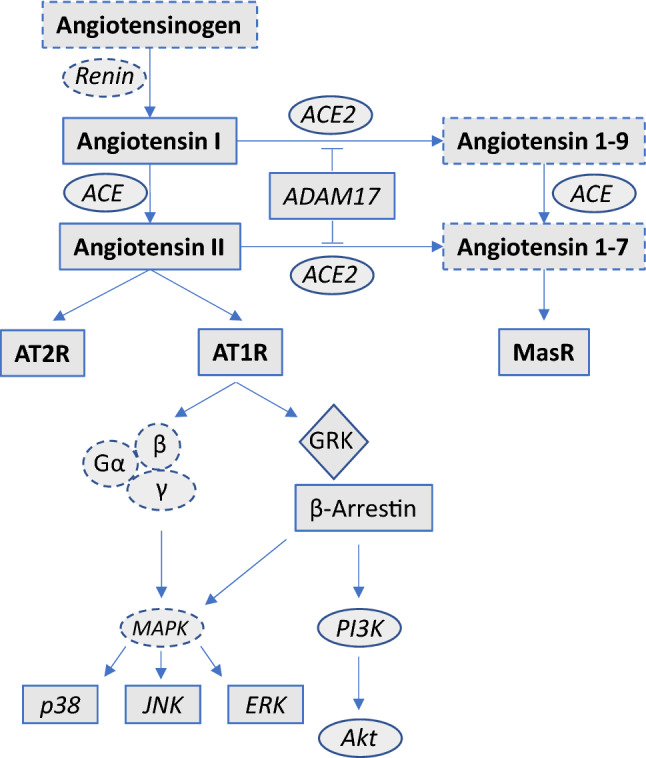
Synopsis of the RAS-β-arrestin signaling pathways. Solid lines: analyzed elements. Dashed lines: elements, which were not analyzed

Due to a further increasing life expectancy and as a clear consequence of improving therapeutic options, the prevalence of patients suffering from end-stage heart failure will continue to increase [1]. Even though heart transplantation is regarded as best therapeutic option for these patients, the ongoing shortage of donor organs, the growing age of patients, and the accumulating load of relevant comorbidities necessitates an implementation of alternative therapies such as left ventricular assist devices (LVADs) [2]. Consequently, the number of LVAD implantations is increasing steadily [3, 4], and in the meantime most patients receive a LVAD as destination therapy, i.e., as last therapeutic option [4, 5]. From this it can be concluded that there is an increasing interest in delineating structural and molecular left ventricular adaptations which accompany this increasingly important therapeutic modality. Recently, our working group described etiology-specific alterations of the cGMP-PKG signaling pathway induced by LVAD therapy [6], and others found important LVAD-mediated adaptations in further pathophysiologically relevant mechanisms in heart failure including apoptosis [7], calcium handling [8], the immune [9], or sympathetic nervous system [10]. But most notably, there remains a remarkable lack of information regarding the most central neurohumoral system, which is activated in progressive heart failure—the renin angiotensin system (RAS). This is all the more striking as there is accumulating evidence that the RAS, which exists as a circulating and tissue-based system, has an increasingly recognized complex and pathophysiologically relevant structure, which not only consists of the known and detrimental ACE/Angiotensin II/AT1R-mediated signaling pathway, but also of a counterbalancing and beneficial ACE2/Angiotensin 1–7/MasR-mediated part [11] (see Fig. 1). Usually, signaling of G protein-coupled receptors (GPCRs) such as the AT1R or AT2R is terminated by GPCR kinase (GRK)- mediated β-arrestin binding to the cytoplasmic receptor loops, which mediates receptor desensitization and internalization. Recent evidence now shows that this signaling pathway is likewise more complicated, as β-arrestins obviously unfold additional and rather protective signaling capacities through multiple downstream mediators, which is why selective activation of β-arrestins became an interesting new therapeutic target [12]. The effects of cardiac unloading by LVAD on this signaling pathway is unknown too.

We, therefore, comprehensively analyzed adaptations of key mediators of the RAS and the known downstream effectors of the β-arrestin signaling pathway in failing left ventricles before and after cardiac unloading by LVAD therapy. We furthermore strove to delineate whether these adaptations are influenced by other determinants such as the heart failure etiology or the duration of LVAD therapy.

## Methods

This study was conducted according to the declaration of Helsinki and was approved by the local ethics committee. All participants gave a written informed consent.

### Study population

Eight patients with end-stage ischemic cardiomyopathy (ICM) and twelve patients with end-stage dilated cardiomyopathy (DCM) who were supported with a LVAD in a bridge to transplant (BTT) intention were included in this study. Both at LVAD implantation and at receiver heart explantation during the heart transplantation procedure, tissue specimens were acquired from left ventricles, respectively, ensuring not to sample scar or fibrotic tissue. Tissue specimens were then instantaneously deep-frozen in liquid nitrogen and stored at − 80 °C.

### Real-time quantitative PCR

Human heart tissue pieces were transferred to a Precellys Lysing Kit Cup (Bertin Technologies, France) with TriFast (Peqlab, Germany) and shredded with a FastPrep-24 homogenizer (MP Biomedicals, USA). After the addition of chloroform, the uppermost aqueous phase contains the RNA, which was added to a new cup containing isopropanol to precipitate a pellet.

The pellet was washed with − 20 °C ethanol (70%), dried, and dissolved in RNAse-free water.

The RNasy Mini Kit and RNase-Free DNase Set (Qiagen, Germany) were used for the DNAse digestion following the manufacturer’s instructions.

Nucleic Acid Concentration measurements were taken with the NanoDrop 2000c Spectrophotometer (ThermoFisher Scientific, USA) at a wavelength of 260 nm.

The cDNA synthesis was performed with the M-MLV Reverse Transcriptase (Promega, USA) according to the manufacturer’s instructions.

Primers were ordered by ThermoFisher Scientific (USA) using the TaqMan assay and the quantitative real-time PCR was performed on the ViiA 7 real-time PCR system (ThermoFisher Scientific, USA) using the ViiA 7 Software (Applied Biosystems, USA). There was a threefold determination for each target:

AT1R (Hs00258938_m1), AT2R (Hs02621316_s1), ACE (Hs00174179_m1), ACE2 (Hs01085333_m1), MasR (Hs00267157_s1), ADAM17 (Hs01041915_m1), and HPRT (HS02800695_m1).

The relative quantification of gene expression was performed using the standard curve method. When using the standard curve method, the quantity of each experimental sample is first determined using a standard curve and is then expressed relative to a calibrator sample. In order to use this quantification method, all samples of interest were pooled and from this four tenfold serial dilutions of cDNA template known to express the gene of interest were prepared. Each serial dilution was used in separate real-time reactions, and their threshold cycle (Ct) values were determined. The Ct values were plotted versus the dilution factor and the data fitted to a straight line. This plot was then used as a standard or calibration curve for extrapolating relative expression level information for the same gene of interest in unknown experimental samples. The relative quantification calibration curve result for the gene of interest was normalized to that of a housekeeping gene in the same sample, and then the normalized numbers are compared between samples to get a fold change in expression. A standard or calibration curve was generated separately for each gene of interest and the used housekeeping gene HPRT.

### Western blot

The tissue samples were pulverized, mixed with lysis buffer, mechanically crushed, and repeatedly cooled in liquid nitrogen. The samples were incubated on ice for 30 min, vortexed for 5–10 min, and the supernatant was transferred to a new cup after centrifugation. The protein concentration was determined by the Pierce BCA Protein Assay Kit (ThermoFisher Scientific, USA) according to the manual instructions. The absorbance was measured by the Infinite M200 Pro Plate Reader (Tecan, Switzerland) at 540 nm.

The protein concentration of each sample was set to 1 μg /μl, consisting of 20% Blue Buffer (+ 10% β-mercaptoethanol), the lysate ,and Dulbecco’s phosphate buffered saline (PBS) (Sigma-Aldrich, Germany). To specifically detect membrane proteins, the samples were denatured at 37 °C for 30 min and cytosolic proteins were denatured at 95 °C for 5 min.

The polymerized gels consist of an 8% SDS-separating gel and a 5% SDS-stacking gel. Electrophoresis took place at 60 mA for about 2 h and a wet transfer at 400 mA for 2 h.

To block unspecific proteins, the membranes were incubated with 5% milk in TBST for 1 h. The primary antibody was diluted in 5% milk in TBST and incubated at 4 °C overnight.

The antibodies were ordered as follows:

ThermoFisher, USA: Anti-PIK3CA (MA5-14870), Anti-ERK1/2 (MA5-15134), Anti-pERK1/2 (700012), Anti-p38 (702273), Anti- pp38 (MA5-15218), Anti-JNK3 (MA5-15403), Anti-pJNK1/2/3 (PA5-36753), Anti-GRK2 (PA5-27480), Anti-β-Arrestin2 (PA1-732) and BD Biosciences, USA: Anti-Akt (610876), Anti-Akt pS473 (560397), and Sigma-Aldrich, Germany: Anti-GAPDH (G8795).

The secondary antibody was diluted in 5% milk in TBST and incubated for 1 h at room temperature. Antibodies were purchased from GE Healthcare, UK: Anti-Rabbit ECL IgG (whole Ab) HRP-linked (sheep) (NA934V) and Anti-Mouse ECL IgG (whole Ab) HRP-linked (sheep) (NA931VS).

The membranes were visualized with the WesternBright ECL (Advansta, USA), according to the provided protocol. Development was performed with the Super-XR FuJi X-ray films (Fujifilm, Germany) and the M35 X-OMAT Processor (Kodak, USA). The exposed X-ray films were scanned with the ChemiDoc MP Imaging System (Bio-rad, USA) and the degrees of blackening of the bands were determined with the ImageJ program.

The blackening of the background was subtracted from the band of the sample and normalized to GAPDH. Normalization to GAPDH acts as a protein loading control.

### Mass spectrometry

Angiotensin metabolites in cardiac tissue were quantified by Attoquant Diagnostics (Vienna, Austria) as described previously [13]. Frozen cardiac tissue segments (50–90 mg) were homogenized using pestle and mortar under liquid nitrogen. The frozen tissue powder was dissolved at 100 mg/ml in 6 mol/l aqueous guanidinium chloride supplemented with 1% (v/v) trifluoroacetic acid (Sigma-Aldrich) by cooled sonication using a 2 mm microtip (Sonics and Materials, Newton, NJ). Stable isotope-labeled internal standards for individual angiotensin metabolites (AngI, AngII) were added to tissue homogenates at 200 pg/ml. The samples then underwent C-18-based solid-phase extraction and were subjected to LC–MS/MS analysis using a reversed phase analytical column operating in line with a Xevo TQ-S triple quadruple mass spectrometer (Waters). Internal standards were used to correct for peptide recovery of the sample preparation procedure for each analyte in each individual sample. Analyte concentrations were reported in fmol/g and are calculated considering the corresponding response factors determined in appropriate calibration curves in original sample matrix, on condition that integrated signals exceeded a signal-to-noise ratio of 10.

### Statistics

The statistical analysis was performed via GraphPad Prism and SPSS. The diagrams were created with GraphPad PRISM. The paired t-test was used to investigate whether LVAD therapy led to changes in the expression of the individual targets. The unpaired *t*-test was used to compare healthy NF patients with heart sick patients. In this case, the variance equality of the groups was first checked via the Levene test. If the variances were equal, a two-sample *t*-test was used; otherwise, with unequal variance, the Welch test was used instead.

The 2-way ANOVA test was used to investigate whether the groups formed by disease, duration of treatment, and ejection fraction showed a difference in treatment between groups and whether there was an interaction between group effect and time.

An *α*-error of less than 5% was defined as statistically significant (*p* < 0.05).

## Results

### Patient characteristics

Most patients were middle-aged men (age 53 ± 10 years) with a severely impaired left ventricular systolic function (LVEF 19 ± 8%) receiving the guideline-recommended pharmacological and device therapy, when a LVAD system was implanted. The mean duration of mechanical unloading then was 331 ± 317 days, before heart transplantation could be performed. There were no significant differences between patients suffering from DCM as compared to those with ICM (see Table 1).

**Table 1:** Baseline characteristics and time on LVAD

	All patients (*n* = 20)	ICM patients (*n* = 8)	DCM patients (*n* = 12)	*P* value
Age (years)	53 ± 10	56 ± 7	51 ± 12	0.285
Male (%)	17 (85)	7 (88)	10 (83)	0.811
Time on LVAD (days)	331 ± 317	206 ± 152	414 ± 374	0.156
LVEF (%)	19 ± 8	21 ± 8	19 ± 9	0.601
ACE inhibitor (%)	10 (59)	3 (50)	7 (64)	0.612
ARB (%)	1 (6)	0 (0)	1 (9)	0.478
Diuretic (%)	14 (82)	5 (83)	9 (82)	0.942
Beta Blocker (%)	11 (65)	3 (50)	8 (73)	0.380
MRA (%)	10 (59)	3 (50)	7 (64)	0.612
Statin (%)	10 (59)	3 (50)	7 (64)	0.612
ICD	10 (59)	3 (50)	7 (64)	0.612

Age, time on LVAD, and LVEF values are expressed as mean ± standard deviation. *LVAD* left ventricular assist device, *LVEF* left ventricular ejection fraction, *ACE* angiotensin converting enzyme, *ARB* angiotensin receptor blocker, *MRA* mineralocorticoid receptor antagonist, *ICD* implanted cardioverter defibrillator. Baseline data are missing for 1 DCM and 2 ICM patients

### RAS ligands and enzymes: Angiotensin I, Angiotensin II, ACE, ACE2, and ADAM17

**Fig. 2:**
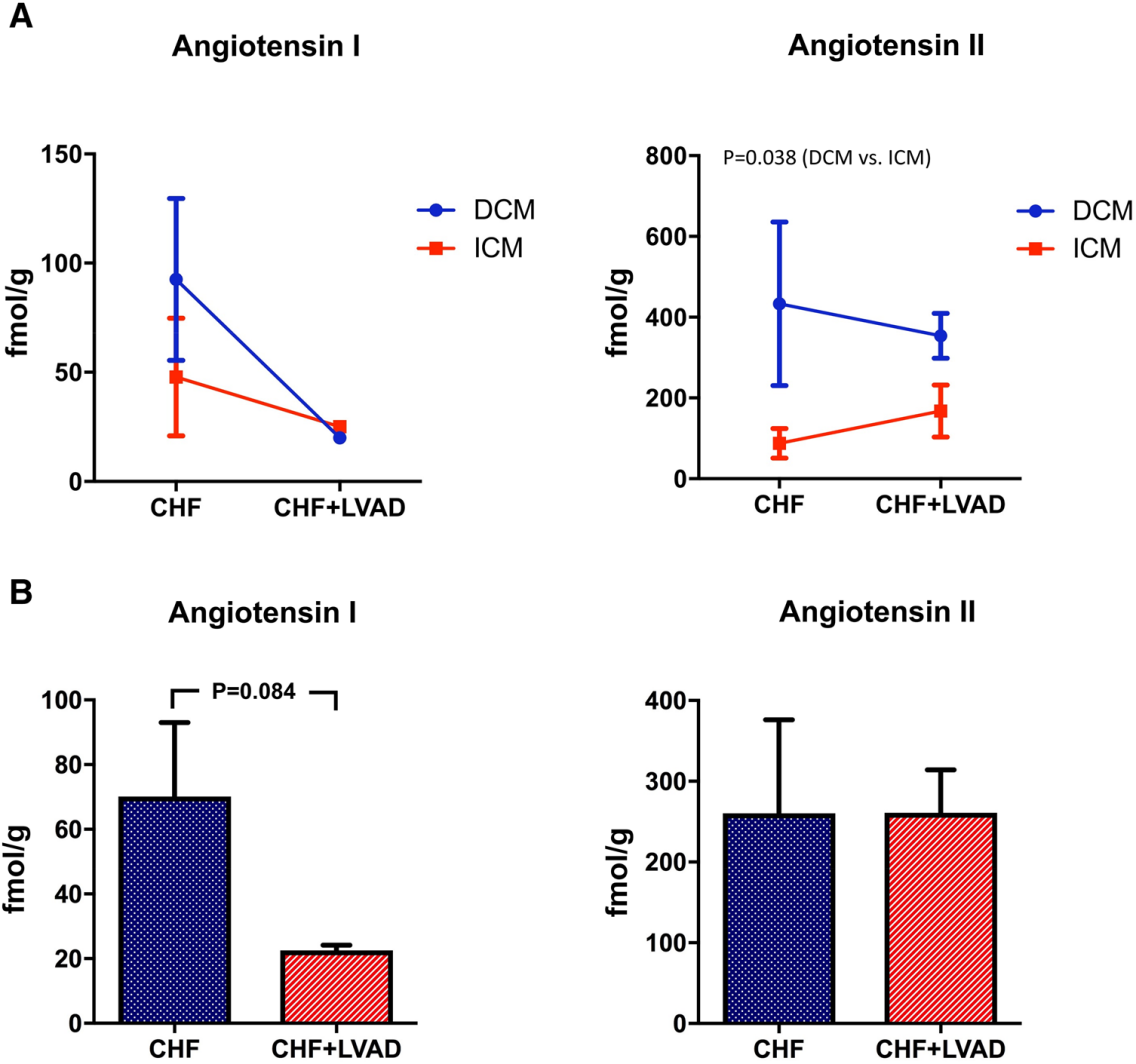
**a** Angiotensin I and Angiotensin II in DCM and ICM before (CHF) and after LVAD therapy (CHF+LVAD). Analyte concentrations were determined by mass spectrometry and are reported here in fmol per gram cardiac tissue. Due to the scarcity of non-failing myocardial tissue specimens, only tissue before and after LVAD support could be analyzed. *CHF* congestive heart failure, *DCM* dilated cardiomyopathy, *ICM* ischemic cardiomyopathy, *LVAD* left ventricular assist device. **b** Angiotensin I and Angiotensin II before (CHF) and after LVAD therapy (CHF+LVAD). Analyte concentrations were determined by mass spectrometry and are reported here in fmol per gram cardiac tissue. Due to the scarcity of non-failing myocardial tissue specimens, only tissue before and after LVAD support could be analyzed. *CHF* congestive heart failure, *DCM* dilated cardiomyopathy, *ICM* ischemic cardiomyopathy, *LVAD* left ventricular assist device

Whereas Angiotensin I expression did not differ between DCM and ICM patients, Angiotensin II was significantly higher expressed in DCM patients (see Fig. 2a). LVAD therapy then reduced Angiotensin I expression (22.54 ± 4.70 vs. 70.12 ± 64.62 AU, *P* = 0.084) irrespective of the heart failure etiology (i.e., DCM vs. ICM) or the time on LVAD. In contrast, Angiotensin II was unaffected by LVAD therapy (see Fig. 2b).

**Fig. 3:**
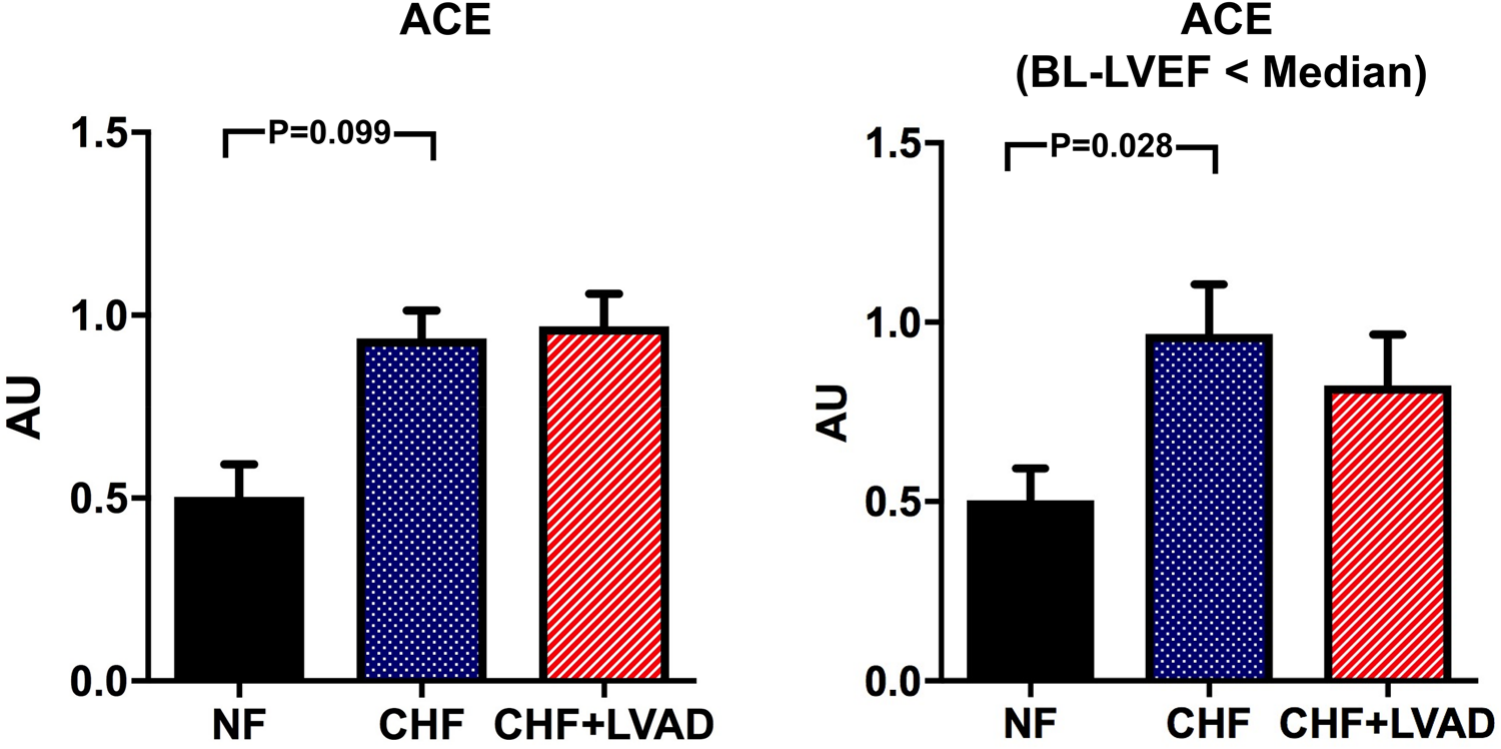
ACE before (CHF) and after LVAD therapy (CHF+LVAD) as compared to non-failing ventricles (NF), left. Same analysis in the subgroup of patients with a baseline left ventricular ejection fraction below the median value, right (*n* = 9). ACE expression was determined by polymerase chain reaction (PCR). The result of each analysis in each sample group was referred to a reference standard, which consisted of a pool of all samples and whose expression level was set 1 by default. The value on the *y*-axis, therefore, reflects the percentage of each parameter's expression level in relation to this default value. *AU* arbitrary unit, *BL-LVEF* baseline left ventricular ejection fraction, *CHF* congestive heart failure, *LVAD* left ventricular assist device, *NF* non-failing myocardial tissue specimen

Angiotensin converting enzyme (ACE) tended to be upregulated in CHF vs. NF (0.94 ± 0.35 vs. 0.50 ± 0.13 AU, *P* = 0.099), which reached statistical significance only when comparing CHF patients with lower vs. higher than median LVEF at baseline (*P* = 0.028) indicating a more pronounced ACE upregulation in more severely impaired left ventricles. In contrast, ACE regulation was not influenced by LVAD therapy (see Fig. 3).

**Fig. 4:**
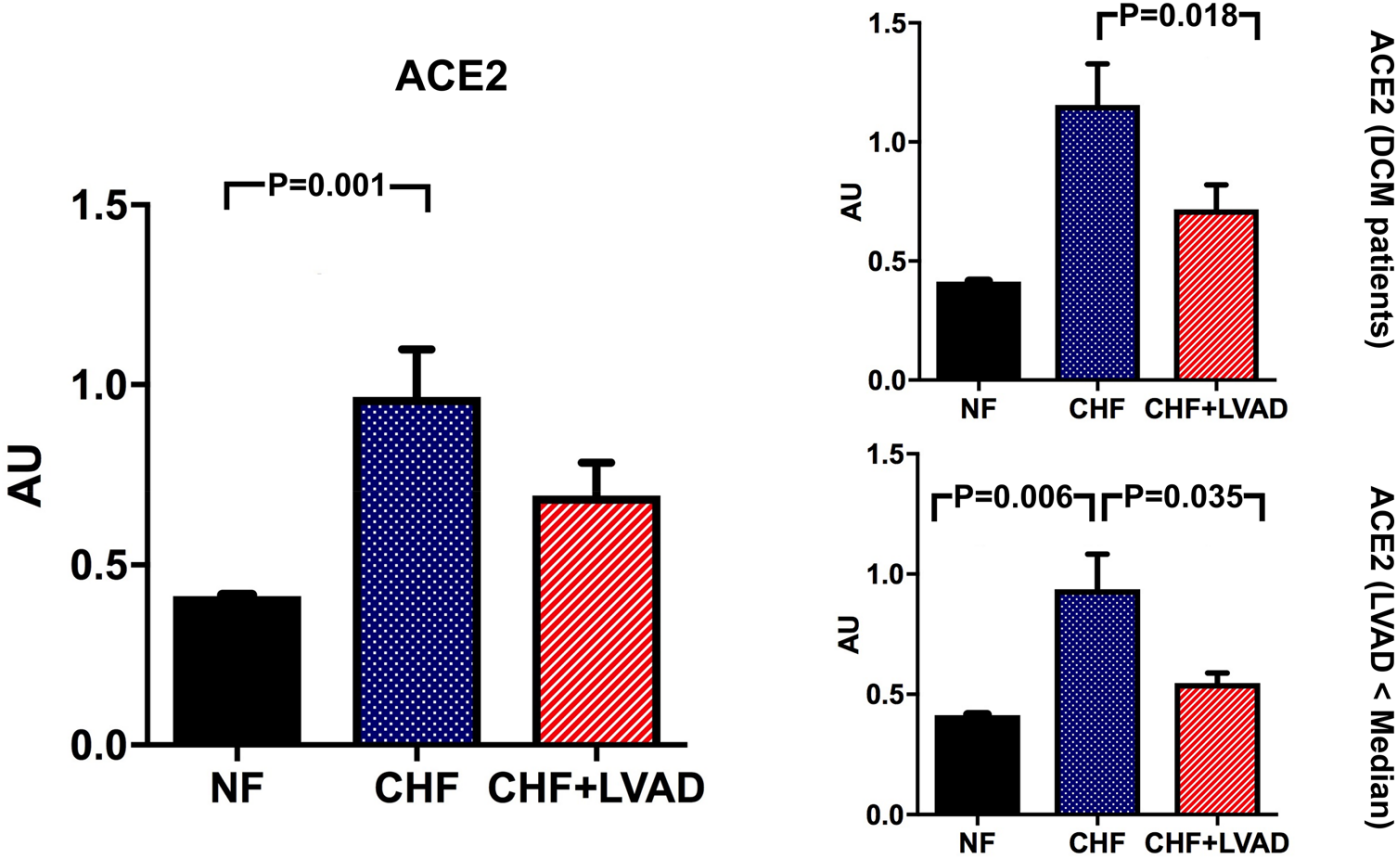
ACE2 before (CHF) and after LVAD therapy (CHF+LVAD) as compared to non-failing ventricles (NF), left. The same analysis for the subgroups of patients with DCM as underlying heart disease (right, above; *n* = 12) and the patients with a duration of LVAD therapy below the median value (right, below; *n* = 10). ACE2 expression was determined by polymerase chain reaction (PCR). The result of each analysis in each sample group was referred to a reference standard, which consisted of a pool of all samples and whose expression level was set 1 by default. The value on the y-axis, therefore, reflects the percentage of each parameter's expression level in relation to this default value. *AU* arbitrary unit, *CHF* congestive heart failure, *DCM* dilated cardiomyopathy, *LVAD* left ventricular assist device, *NF* non-failing myocardial tissue specimen

Likewise, ACE2 was upregulated in CHF vs. NF (0.97 ± 0.59 vs. 0.41 ± 0.004 AU, *P* = 0.001). In contrast to ACE, upregulation of ACE2 was more pronounced in less severely impaired left ventricles (1.10 ± 0.43 vs. 0.41 ± 0.004 AU, *P* = 0.054). Even though LVAD therapy seemed to moderately reduce ACE2 expression, this only reached statistical significance in DCM patients and in patients with shorter time on LVAD therapy (see Fig. 4).

ADAM17, which can cleave ACE2, did not show differential regulation in CHF vs. NF and was not affected by LVAD therapy (data not shown).

### RAS receptors: AT1R, AT2R, MasR

**Fig. 5:**
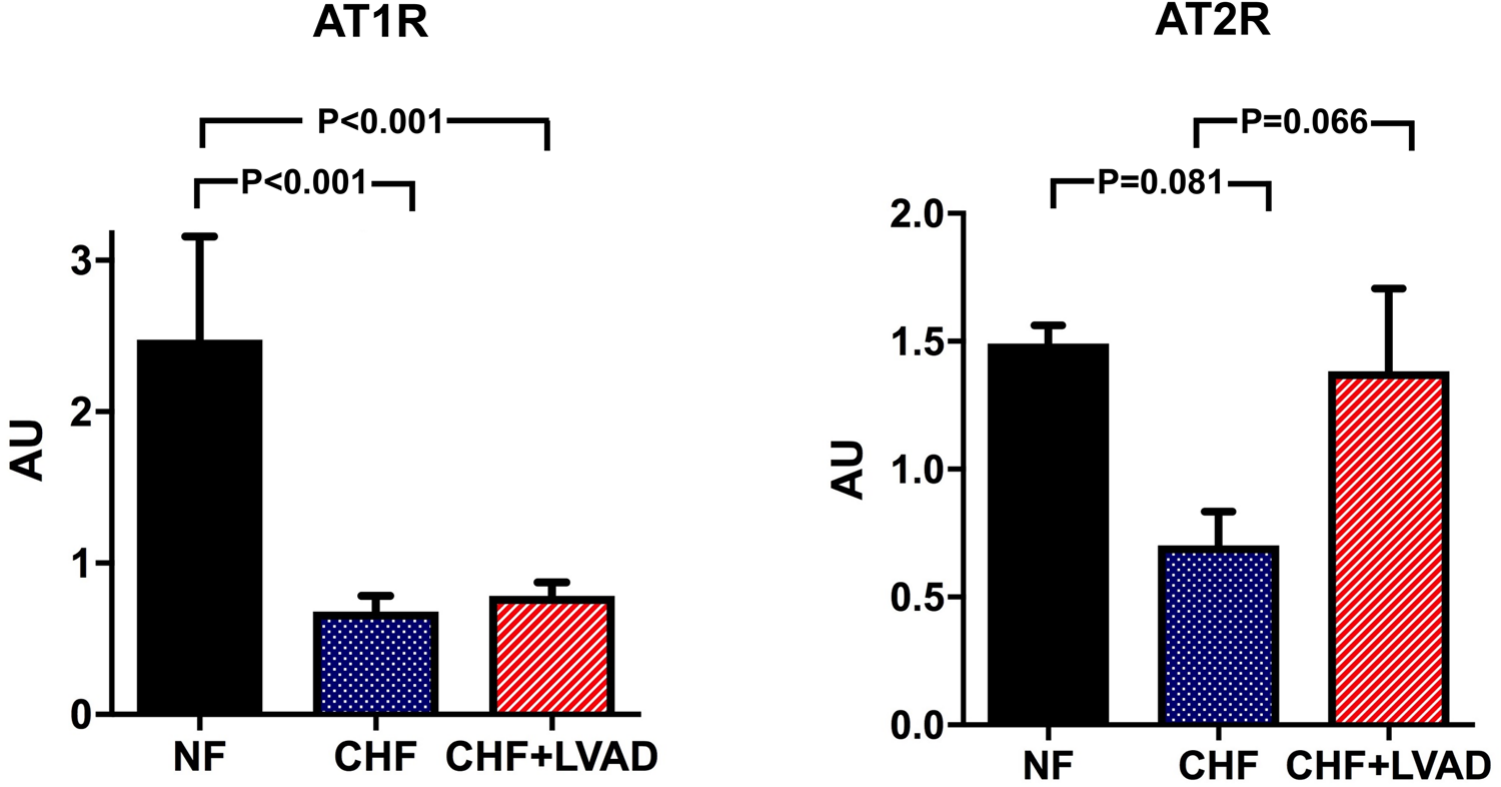
AT1R (left) and AT2R (right) before (CHF) and after LVAD therapy (CHF+LVAD) as compared to non-failing ventricles (NF). AT1R and AT2R expression were determined by polymerase chain reaction (PCR). The result of each analysis in each sample group was referred to a reference standard, which consisted of a pool of all samples and whose expression level was set 1 by default. The value on the *y*-axis, therefore, reflects the percentage of each parameter's expression level in relation to this default value. *AU* arbitrary unit, *CHF* congestive heart failure, *LVAD* left ventricular assist device, *NF* non-failing myocardial tissue specimen

AT1R was significantly downregulated in CHF vs. NF (0.68 ± 0.47 vs. 2.48 ± 0.96 AU, *P* < 0.001), but was further on not altered by LVAD therapy (see Fig. 5). This regulation pattern was similar in ICM and DCM patients and was not influenced by the duration of LVAD therapy.

Even though AT2R tended to be likewise downregulated in CHF as compared to NF (0.70 ± 0.59 vs. 1.49 ± 0.10 AU, *P* = 0.081) predominantly in DCM patients, LVAD therapy caused an upregulation (1.38 ± 1.45 vs. 0.70 ± 0.59 AU, *P* = 0.066), which did not differ between DCM and ICM. Interestingly, this upregulation was more pronounced in left ventricles with a more reduced ejection fraction at baseline (1.96 ± 1.86 vs. 0.90 ± 0.87 AU, *P* = 0.064).

**Fig. 6:**
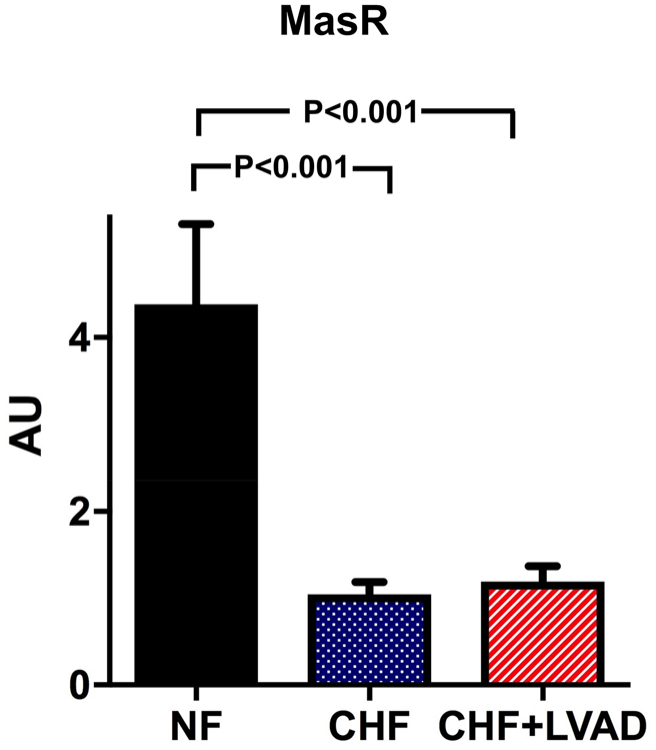
MasR before (CHF) and after LVAD therapy (CHF+LVAD) as compared to non-failing ventricles (NF). MasR expression was determined by polymerase chain reaction (PCR). The result of each analysis in each sample group was referred to a reference standard, which consisted of a pool of all samples and whose expression level was set 1 by default. The value on the *y*-axis, therefore, reflects the percentage of each parameter's expression level in relation to this default value. *AU* arbitrary unit, *CHF* congestive heart failure, *LVAD* left ventricular assist device, *NF* non-failing myocardial tissue specimen

MasR, which serves as a receptor for Angiotensin 1–7, was regulated very similar to AT1R with a significant downregulation in CHF as compared to NF (1.04 ± 0.59 vs. 4.38 ± 1.31 AU, *P* < 0.001) and without any alteration upon LVAD therapy (see Fig. 6).

### GRK2/β-arrestin 2 and downstream targets: MAPK (ERK, p38, JNK) and PI3K/Akt

**Fig. 7:**
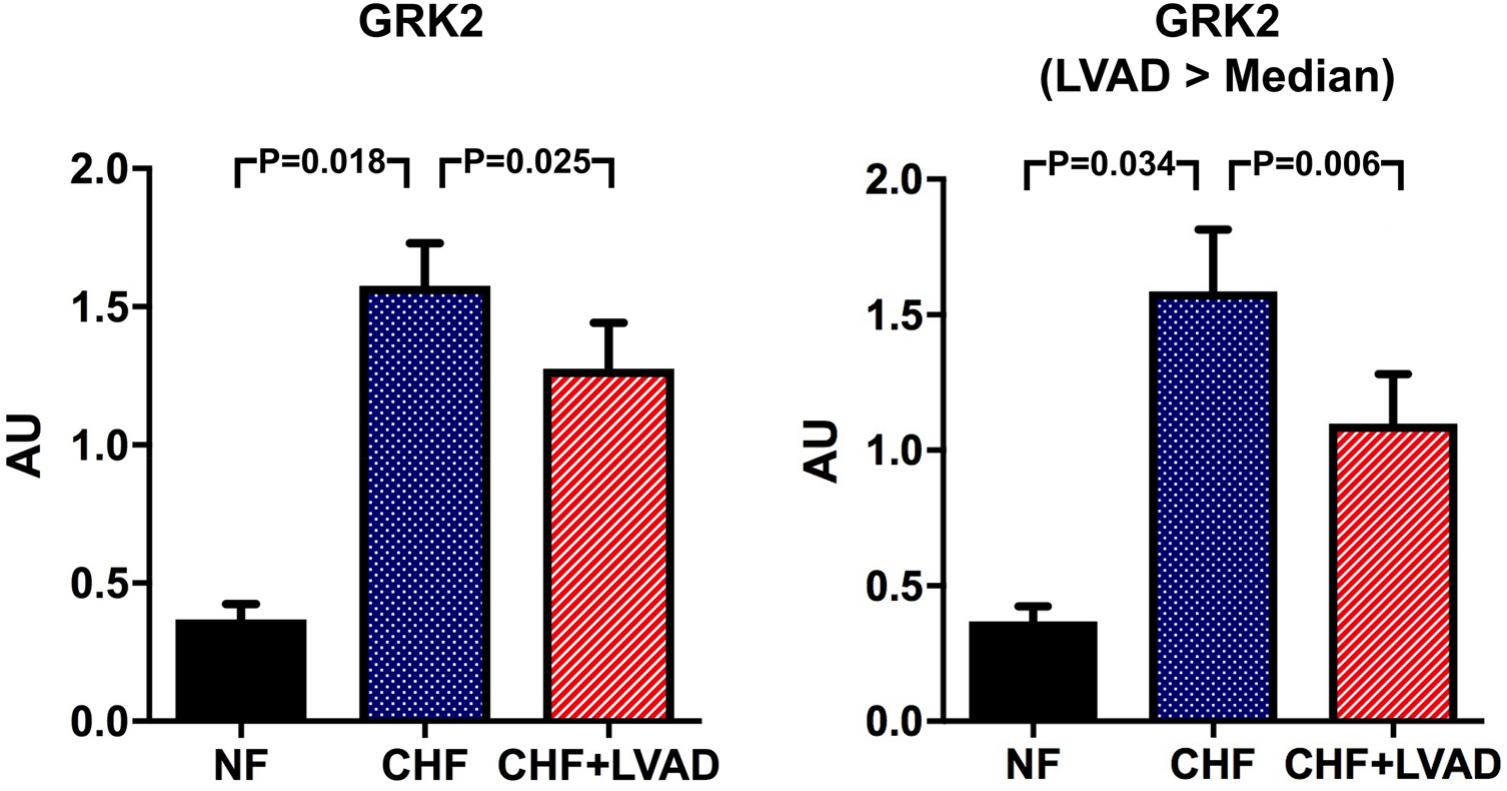
GRK2 before (CHF) and after LVAD therapy (CHF+LVAD) as compared to non-failing ventricles (NF), left. The same analysis for the subgroups of patients with a duration of LVAD therapy above the median value, right (*n* = 10). GRK2 expression was determined by immunoblot (western blot) analysis and referred to a standard, respectively, whose densitometric value was set 1 by default. The value on the *y*-axis, therefore, reflects the percentage of each parameter's immunoblot band density in relation to this default value. *AU* arbitrary unit, *CHF* congestive heart failure, *LVAD* left ventricular assist device, *NF* non-failing myocardial tissue specimen

G protein-coupled receptor kinase 2 (GRK2) was significantly higher expressed in CHF as compared to NF (1.58 ± 0.64 vs. 0.37 ± 0.08 AU; *P* = 0.018). LVAD therapy significantly lowered these increased GRK2 expression levels (1.28 ± 0.68 vs. 1.58 ± 0.64, *P* = 0.025) predominantly in DCM patients and in those treated by LVAD for a longer time (see Fig. 7).

**Fig. 8:**
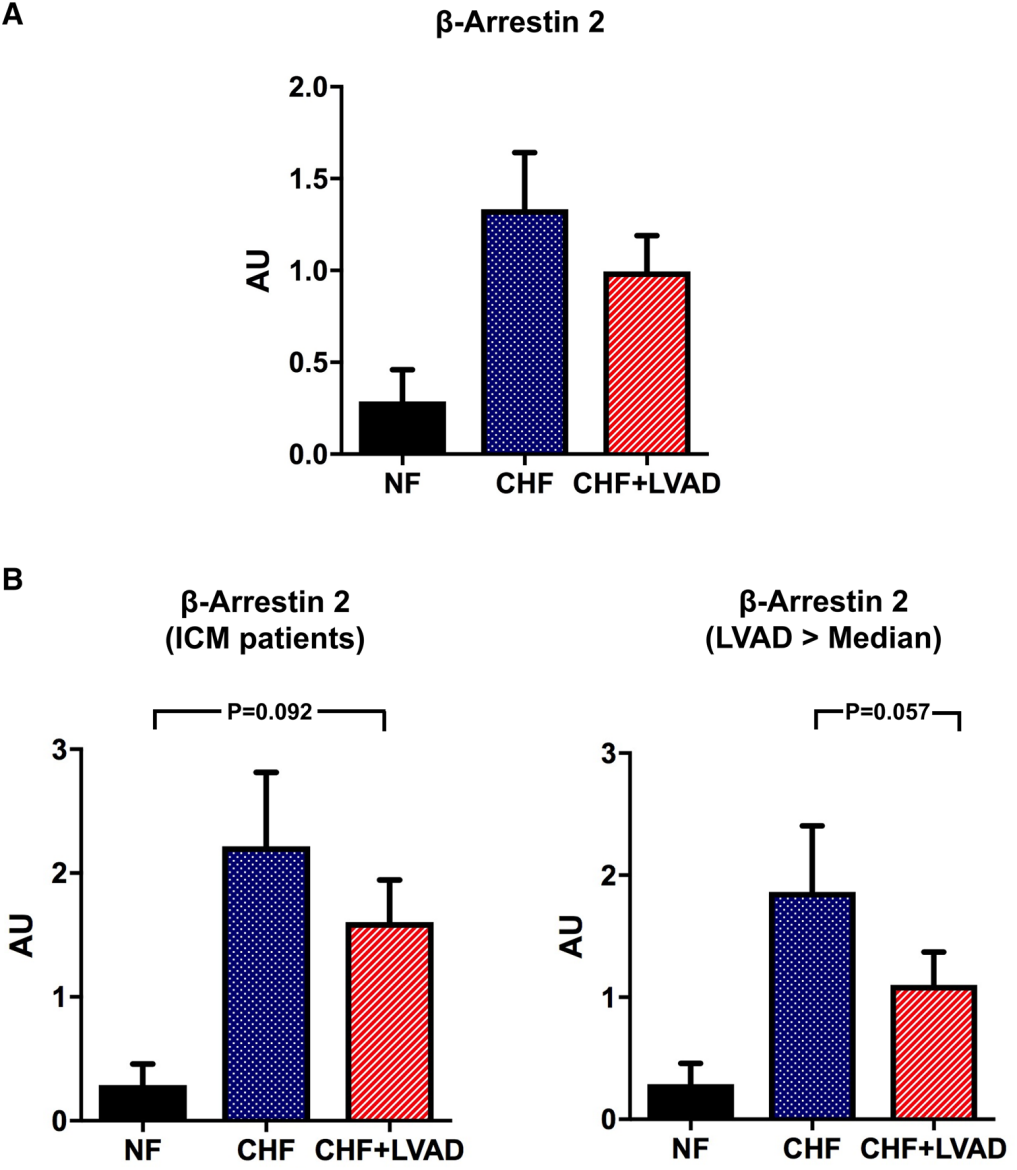
**a** β-arrestin 2 before (CHF) and after LVAD therapy (CHF+LVAD) as compared to non-failing ventricles (NF). β-arrestin 2 expression was determined by immunoblot (western blot) analysis and referred to a standard, respectively, whose densitometric value was set 1 by default. The value on the *y*-axis, therefore, reflects the percentage of each parameter's immunoblot band density in relation to this default value. *AU* arbitrary unit, *CHF* congestive heart failure, *LVAD* left ventricular assist device, *NF* non-failing myocardial tissue specimen. **b** β-arrestin 2 before (CHF) and after LVAD therapy (CHF+LVAD) in the subgroup of ICM patients as compared to non-failing ventricles (NF), left (*n* = 8). β-arrestin 2 before (CHF) and after LVAD therapy (CHF+LVAD) in the subgroup of 10 patients with a duration of LVAD therapy above the median value as compared to non-failing ventricles (NF), right. *AU* arbitrary unit, *CHF* congestive heart failure, *ICM* ischemic cardiomyopathy, *LVAD* left ventricular assist device, *NF* non-failing myocardial tissue specimen

β-arrestin 2 expression tended to be higher both in CHF and after LVAD therapy (see Fig. 8a) particularly in ICM patients (1.60 ± 0.90 vs. 0.46 ± 0.12 AU by comparing CHF+LVAD with NF, *P* = 0.092; see Fig. 8b). LVAD therapy caused a lowering of β-arrestin 2 expression levels in those patients with a longer duration of cardiac unloading (1.10 ± 0.76 vs. 1.86 ± 1.53 AU by comparing CHF+LVAD with CHF, *P* = 0.057), whereas a shorter LVAD duration did not have any impact (see Fig. 8b).

**Fig. 9:**
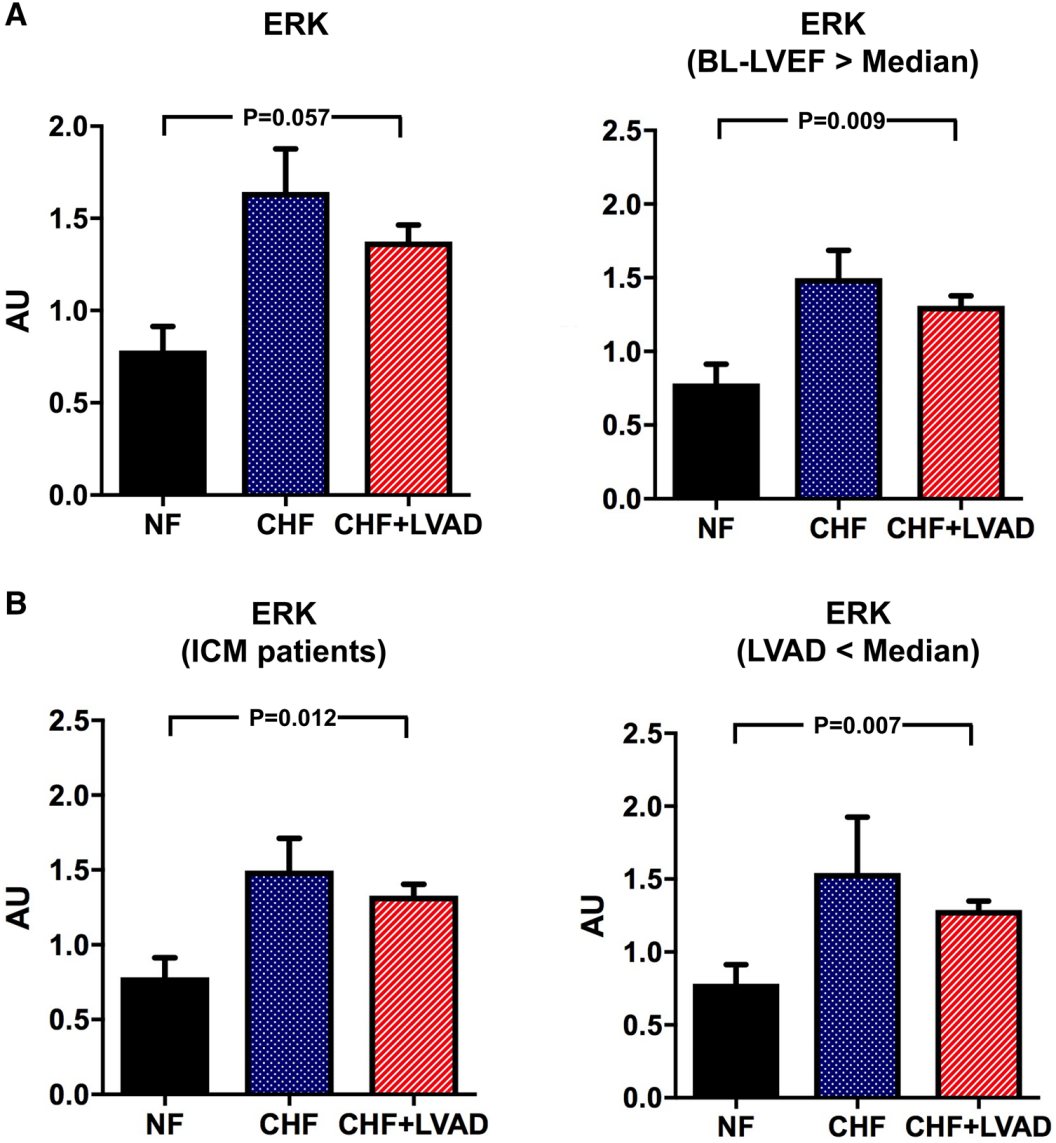
**a** ERK before (CHF) and after LVAD therapy (CHF+LVAD) as compared to non-failing ventricles (NF), left. The same comparisons in the subgroup of patients with a baseline left ventricular ejection fraction above the median value, right (*n* = 10). ERK expression was determined by immunoblot (western blot) analysis and referred to a standard, respectively, whose densitometric value was set 1 by default. The value on the *y*-axis, therefore, reflects the percentage of each parameter's immunoblot band density in relation to this default value. *AU* arbitrary unit, *BL-LVEF* baseline left ventricular ejection fraction, *CHF* congestive heart failure, *LVAD* left ventricular assist device, *NF* non-failing myocardial tissue specimen. **b** ERK before (CHF) and after LVAD therapy (CHF+LVAD) as compared to non-failing ventricles (NF) in the subgroup of ICM patients, left (*n* = 8). The same comparisons in the subgroup of patients with a duration of LVAD therapy below the median value, right (*n* = 10). *AU* arbitrary unit, *CHF* congestive heart failure, *ICM* ischemic cardiomyopathy, *LVAD* left ventricular assist device, *NF* non-failing myocardial tissue specimen

ERK, JNK, and p38 belong to the family of mitogen-activated protein kinases (MAPK) and were analyzed as important downstream targets of GRK2/β-arrestin 2 signaling. ERK tended to be upregulated in CHF patients and after LVAD therapy meeting statistical significance in ICM patients (1.39 ± 0.47 vs. 0.72 ± 0.20 AU, *P* = 0.012 by comparing CHF+LVAD vs. NF) and in those treated for a shorter time with LVAD (1.29 ± 0.20 vs. 0.72 ± 0.20 AU, *P* = 0.007 by comparing CHF+LVAD vs. NF) and with a higher LVEF at baseline (1.31 ± 0.21 vs. 0.72 ± 0.20 AU, *P* = 0.009 by comparing CHF+LVAD vs. NF; see Fig. 9a and b). Phosphorylated ERK in turn did not show any change in heart failure or after LVAD therapy.

**Fig. 10:**
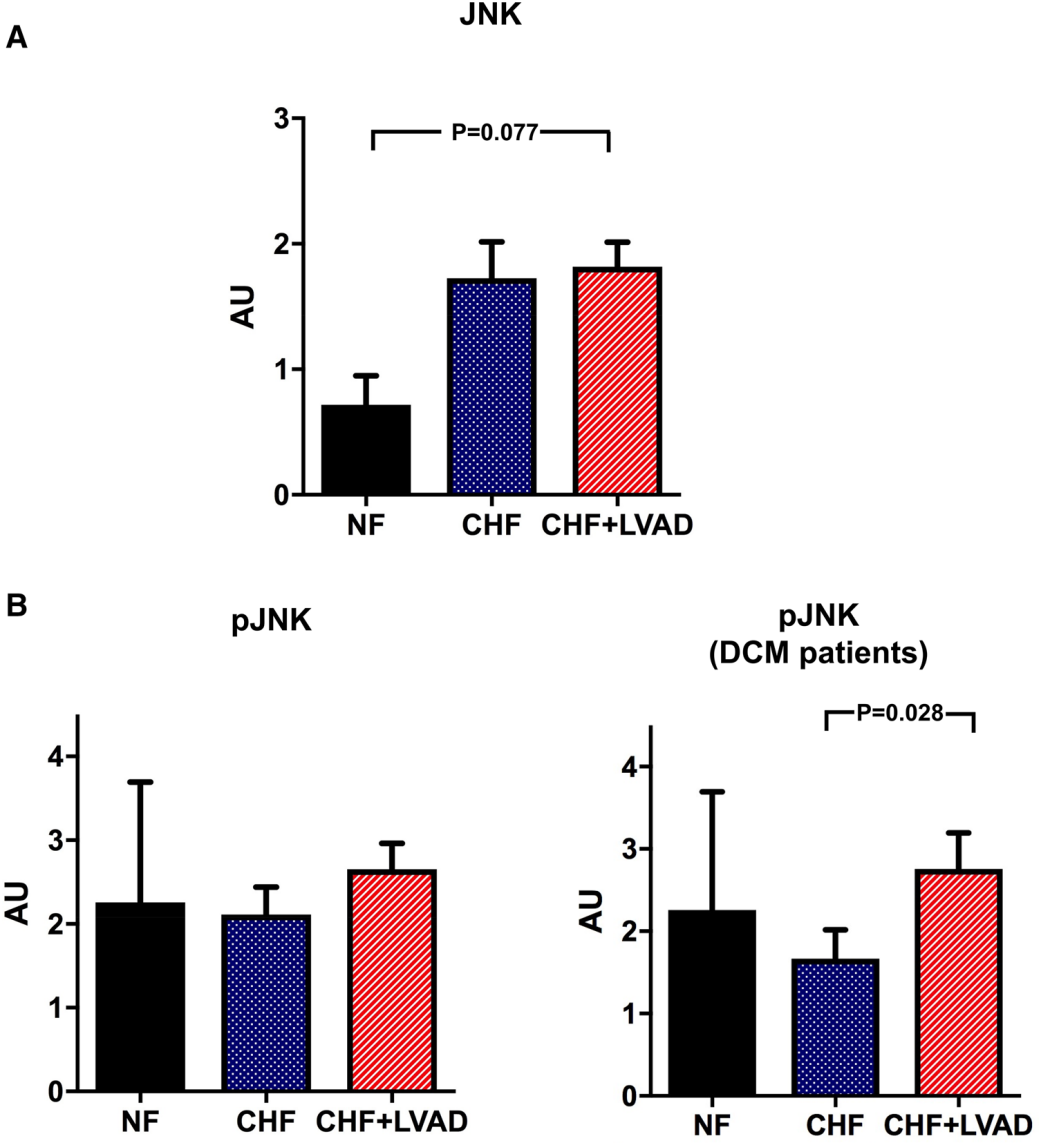
**a** JNK before (CHF) and after LVAD therapy (CHF+LVAD) as compared to non-failing ventricles (NF). JNK expression was determined by immunoblot (western blot) analysis and referred to a standard, respectively, whose densitometric value was set 1 by default. The value on the *y*-axis, therefore, reflects the percentage of each parameter's immunoblot band density in relation to this default value. *AU* arbitrary unit, *CHF* congestive heart failure, *LVAD* left ventricular assist device, *NF* non-failing myocardial tissue specimen. **b** Phosphorylated JNK (pJNK) before (CHF) and after LVAD therapy (CHF+LVAD) as compared to non-failing ventricles (NF), left. The same comparisons in the subgroup of DCM patients, right (*n* = 12). *AU* arbitrary unit, *CHF* congestive heart failure, *DCM* dilated cardiomyopathy, *LVAD* left ventricular assist device, *NF* non-failing myocardial tissue specimen

Similarly, JNK tended to be upregulated in CHF and after LVAD therapy (see Fig. 10a), even though statistical significance was only met for phosphorylated JNK in DCM patients (2.76 ± 1.52 vs. 1.57 ± 1.21 AU, *P* = 0.028 by comparing CHF+LVAD vs. CHF; see Fig. 10b).

**Fig. 11:**
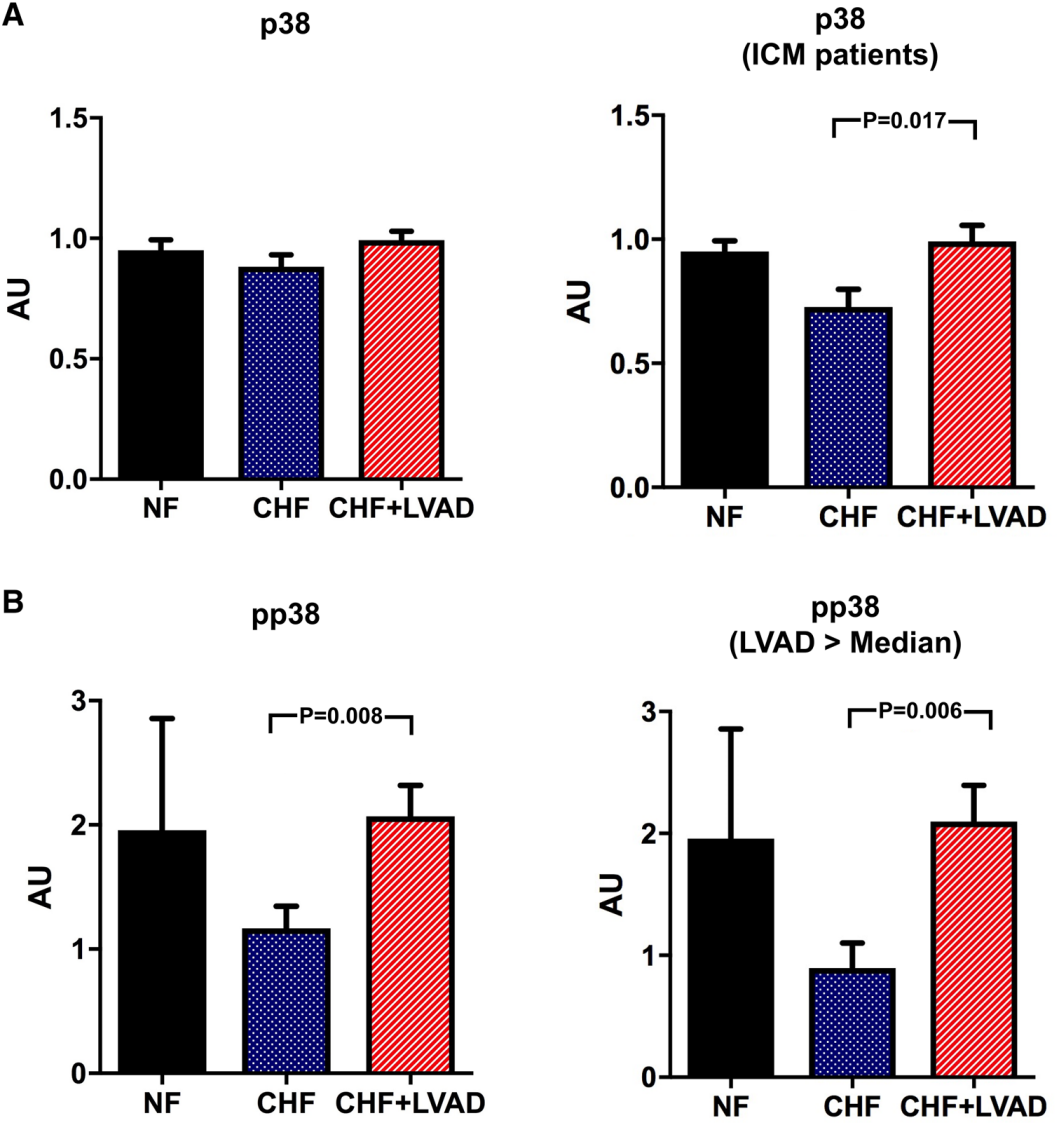
**a** p38 before (CHF) and after LVAD therapy (CHF+LVAD) as compared to non-failing ventricles (NF), left. The same comparisons in the subgroup of ICM patients, right (*n* = 8). P38 expression was determined by immunoblot (Western blot) analysis and referred to a standard, respectively, whose densitometric value was set 1 by default. The value on the *y*-axis, therefore, reflects the percentage of each parameter's immunoblot band density in relation to this default value. *AU* arbitrary unit, *CHF* congestive heart failure, *ICM* ischemic cardiomyopathy, *LVAD* left ventricular assist device, *NF* non-failing myocardial tissue specimen. **b** Phosphorylated p38 (pp38) before (CHF) and after LVAD therapy (CHF+LVAD) as compared to non-failing ventricles (NF), left. The same comparisons in the subgroup of patients with a duration of LVAD therapy above the median value, right (*n* = 10). *AU* arbitrary unit, *CHF* congestive heart failure, *LVAD* left ventricular assist device, *NF* non-failing myocardial tissue specimen

In contrast, p38 showed a significant upregulation only in ICM patients (0.99 ± 0.18 vs. 0.73 ± 0.20 AU, *P* = 0.017 by comparing CHF+LVAD vs. CHF) and was not influenced by baseline LVEF and duration of LVAD therapy (see Fig. 11a). Phosphorylated p38 was in turn significantly upregulated after LVAD therapy irrespective of heart failure etiology (2.07 ± 1.11 vs. 1.17 ± 0.80 AU, *P* = 0.008 by comparing CHF+LVAD vs. CHF) especially in those with a longer time on LVAD (2.10 ± 0.94 vs. 0.90 ± 0.65 AU, *P* = 0.006 by comparing CHF+LVAD vs. CHF) and a lower LVEF at baseline (2.11 ± 1.19 vs. 0.80 ± 0.43 AU, *P* = 0.021 by comparing CHF+LVAD vs. CHF; see Fig. 11b).

**Fig. 12:**
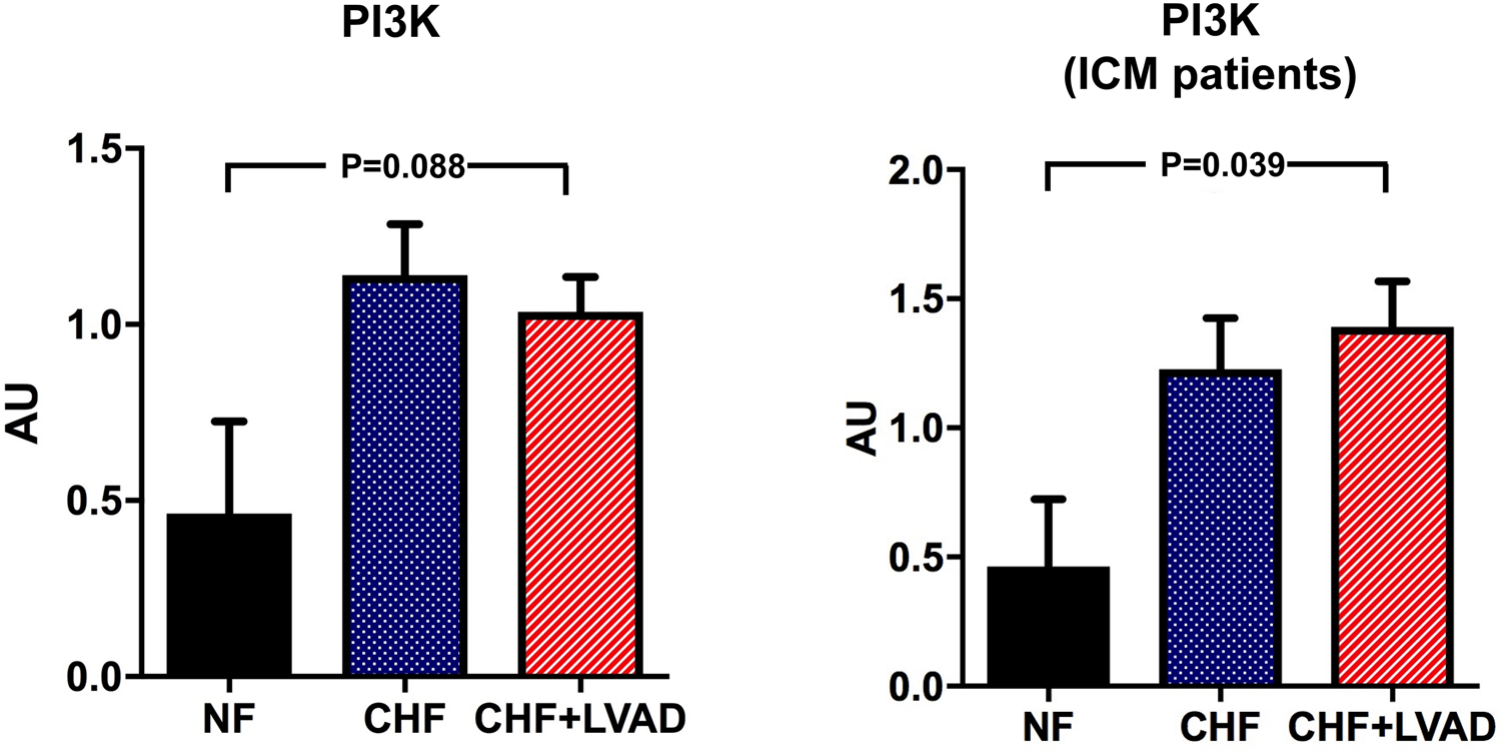
PI3K before (CHF) and after LVAD therapy (CHF+LVAD) as compared to non-failing ventricles (NF), left. The same comparisons in the subgroup of ICM patients, right (*n* = 8). PI3K expression was determined by immunoblot (western blot) analysis and referred to a standard, respectively, whose densitometric value was set 1 by default. The value on the *y*-axis, therefore, reflects the percentage of each parameter's immunoblot band density in relation to this default value. *AU* arbitrary unit, *CHF* congestive heart failure, *ICM* ischemic cardiomyopathy, *LVAD* left ventricular assist device, *NF* non-failing myocardial tissue specimen

**Fig. 13:**
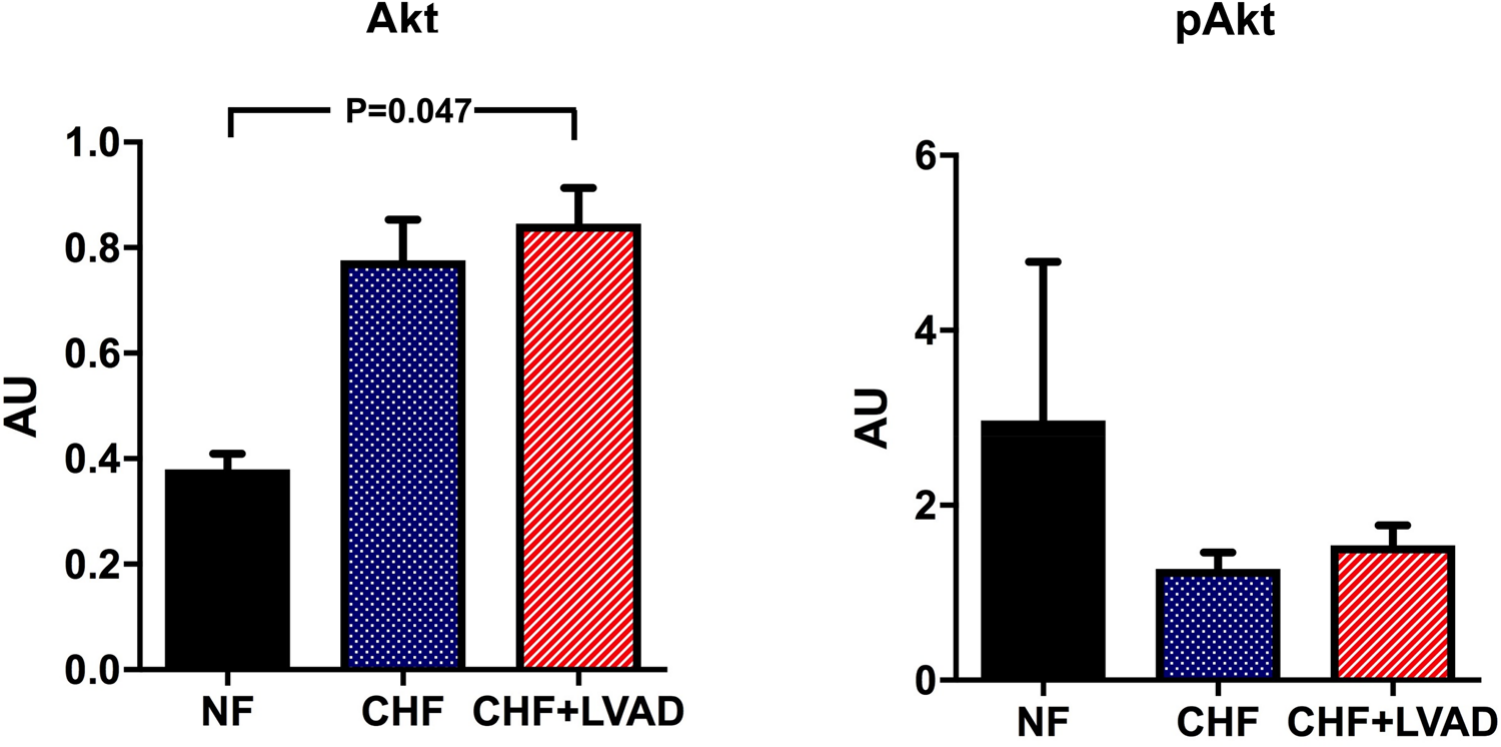
Akt (left) and phosphorylated Akt (pAkt, right) before (CHF) and after LVAD therapy (CHF+LVAD) as compared to non-failing ventricles (NF). Akt and pAkt expression was determined by immunoblot (western blot) analysis and referred to a standard, respectively, whose densitometric value was set 1 by default. The value on the *y*-axis, therefore, reflects the percentage of each parameter's immunoblot band density in relation to this default value. *AU* arbitrary unit, *CHF* congestive heart failure, *LVAD* left ventricular assist device, *NF* non-failing myocardial tissue specimen

Finally, the PI3K/Akt pathway was analyzed as downstream target of GRK/β-arrestin signaling. PI3K was significantly upregulated in LVAD-treated ICM patients (1.39 ± 0.47 vs. 0.46 ± 0.37 AU, *P* = 0.039 by comparing CHF+LVAD vs. NF; see Fig. 12). Similarly, Akt showed a significant upregulation after LVAD therapy (0.85 ± 0.30 vs. 0.38 ± 0.04, *P* = 0.047 by comparing CHF+LVAD vs. NF), which was otherwise irrespective of heart failure etiology. Phosphorylated Akt in turn was not significantly altered in heart failure and after LVAD therapy (see Fig. 13).

**Fig. 14:**
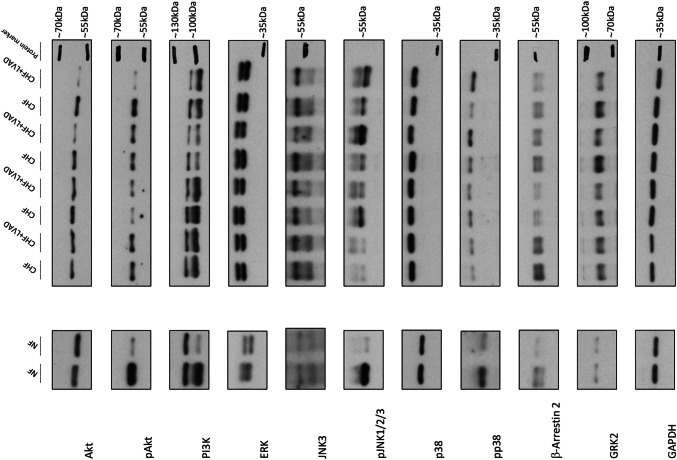
Immunoblot images of the analyzed targets. CHF (congestive heart failure) denotes left ventricles before LVAD therapy. CHF+LVAD (left ventricular assist device) denotes left ventricles after LVAD therapy. The bands of all samples were normalized to GAPDH

**Fig. 15:**
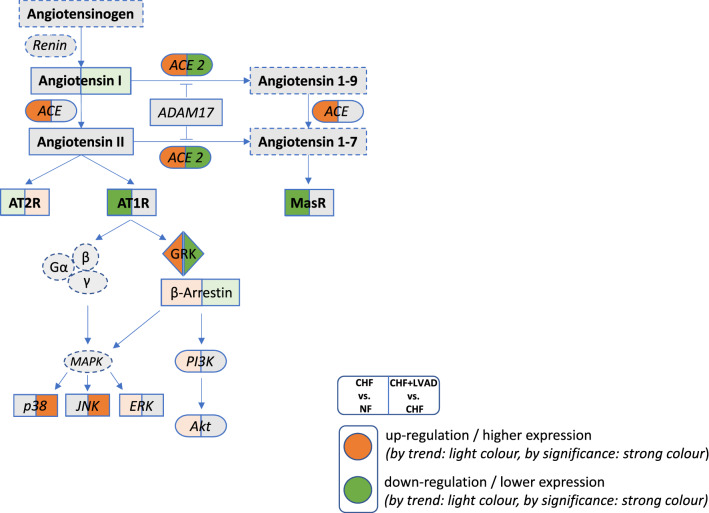
Synopsis of the alterations in failing left ventricles (CHF) vs. non-failing left ventricles (NF; left part of each field) and in LVAD-treated left ventricles (CHF+LVAD) vs. non-LVAD-treated failing left ventricles (CHF; right part of each field). Orange color denotes upregulation or higher expression (solid: significant regulation, light: insignificant trend). Green color denotes downregulation or lower expression (solid: significant regulation, light: insignificant trend). (Color figure online)

Figure 14 displays immunoblot images of the analyzed targets, and Fig. 15 synoptically summarizes the described alterations in CHF and after LVAD therapy.

## Discussion

Our work analyzed adaptations of key mediators of the cardiac renin angiotensin system and the associated β-arrestin signaling pathway in failing left ventricles before and after LVAD therapy and yielded the following main results:

1. Key components of the RAS and the β-arrestin signaling pathways were divergently altered in end-stage heart failure with a downregulation of RAS receptors (i.e., AT1R, AT2R, MasR) and an upregulation of many other upstream and downstream located pathway components (i.e., ACE, ACE2, GRK, β-arrestin, ERK, PI3K, Akt).
2. LVAD therapy then had a complex and highly heterogeneous effect with a down- (angiotensin I, ACE2, GRK, β-arrestin), up- (AT2R, JNK, p38), or unchanged regulation pattern (ACE, angiotensin II, AT1R, ADAM17, MasR, ERK, PI3K, Akt).
3. Some of these expression changes or regulation patterns depend on the etiology of heart failure (i.e., ICM vs. DCM), the severity of heart failure before LVAD implantation, and the duration of LVAD therapy.

### Regulation of components of the RAS and β-arrestin signaling pathways in end-stage heart failure

Our finding of a downregulation of RAS receptors in end-stage heart failure well corroborates the available literature [14–17], even though some controversy remains regarding AT2R expression changes [14, 17]. Our work found a small, but statistical insignificant downregulation of AT2R predominantly in DCM patients, which rather supports the results reported by Regitz-Zagrosek et al. [15], than by Asano et al. [14]. One possible explanation for this could be the differing severity of heart failure, since Regitz-Zagrosek et al. found a loss of AT2R only in end-stage, but not in moderate heart failure [15]. This assumption is also supported by the animal study of Dias-Peixoto et al., who showed a downregulation of MasR only in later, but not in earlier stages after myocardial infarction [16] indicating that a downregulation of RAS receptors might be a characteristic of progressive heart failure.

In contrast, however, important up- (such as ACE, ACE2) and downstream targets (such as GRK, β-arrestin, ERK, PI3K, Akt) of the RAS were higher expressed or upregulated in terminal heart failure. It would be tempting to speculate that this regulation pattern aims at compensating or overcoming the downregulation of RAS receptors at both the up- and downstream sites in order to maintain the overall signaling capacity. This would particularly apply to the protective ACE2/Ang1-7/MasR pathway, since only ACE2, but not ACE expression was significantly altered in the complete patient cohort of our work. A similar finding was reported by Ferrario et al. in their animal study, where an angiotensin receptor blockade using losartan augmented ACE2, but not ACE mRNA expression [18]. Having said this, it is very interesting to then find a significant upregulation of ACE exclusively in the subgroup of patients with the lowest left ventricular ejection fraction at baseline in our study, since this could indicate a strengthening of the detrimental ACE-mediated axis, which counterbalances the activated and protective ACE2-mediated site of the RAS as soon as heart failure has reached its most advanced stages.

### Effects of cardiac unloading by LVAD on components of the RAS and β-arrestin signaling pathways

The impact of LVAD therapy is well described for structural (e.g., effects on myocyte size [19]) or the composition of cytoskeletal proteins [20] and the extracellular matrix [21]) and many molecular alterations (e.g., effects on metabolic enzymes [22] or components of the immune [9] and the sympathetic nervous system [10]).

Furthermore, genomic profiling revealed a LVAD-mediated alteration of several gene sets, such as genes involved in cell growth, apoptosis and cell signaling or genes controlling for the formation of vascular networks and the expression of diverse transcription factors [23, 24]. Interestingly, one of these transcription factors, i.e., Forkhead box transcription factor FOX03A, resembled the expression patterns of Angiotensin II type 1 receptor before and after LVAD support, thus indicating a mechanistic link [24]. But besides these investigations there have been only very few studies so far focusing on the pathophysiologically central RAS and β-arrestin signaling pathways or at least parts of it: Welp et al. compared the plasma renin activity and plasma aldosterone levels of patients, who were treated with either pulsatile or non-pulsatile devices [25], and Klotz et al. analyzed the effects of LVAD therapy with and without concomitant ACE inhibitor medication on cardiac renin, aldosterone and norepinephrine expression levels [26]. Neither study evaluated further up- or downstream components of the renin angiotensin system.

This was partly done by Baba et al., who focused on phosphorylation patterns of mitogen-activated protein kinases (e.g., ERK, JNK, p38) and of the anti-apoptotic kinase Akt [27]. In this work, a strong decrease of phosphorylated ERK-1, ERK-2, and Akt was found after LVAD therapy, whereas the phosphorylation patterns of JNK and p38 remained unchanged. Another study by Razeghi et al. described a likewise decrease of ERK phosphorylation levels after LVAD support, but could not verify any phosphorylation changes within the Akt pathway [28]. In contrast to these analyses, our work showed no influence of LVAD therapy on either total or phosphorylated ERK and Akt levels, only phosphorylated p38 was significantly upregulated after LVAD support. These discrepancies cannot be easily explained. Most probably, they are due to the highly heterogeneous patient cohorts under investigation, where enzyme expression and phosphorylation patterns might be influenced by many other, poorly controllable determinants than by the LVAD therapy alone (such as comorbidities, medications, or the underlying heart failure etiology). Even the duration of LVAD support could have been relevant (as we could show for other parameters; see next paragraph), since the mean time on LVAD was remarkably longer in our study (331 days) than in Baba's (222 days) or Razeghi's (205 days) works.

Beyond these single targets and in consideration of the many other parameters, which we determined in our study, we generally found a rather heterogenous LVAD-mediated effect on the RAS and β-arrestin signaling pathway with a downregulation of angiotensin I, ACE2, GRK, β-arrestin, an upregulation of AT2R, JNK, p38 and an unchanged regulation pattern of angiotensin II, ACE, ADAM17, AT1R, MasR, ERK, PI3K and Akt. Notably, most of these expression changes at least partly occurred in a direction, which aimed at restoring the expression patterns of non-failing left ventricles. This supports and extends the existing evidence, which clearly demonstrates a positive effect of cardiac unloading on many pathophysiologically relevant pathways in heart failure, as it was excellently gathered by Birks [29]. But on the other hand, our work also showed that this molecular reverse remodeling is remarkably incomplete, since many components of both the detrimental ACE/Angiotensin II/AT1R- and the beneficial ACE2/Ang1-7/MasR- axis of the renin angiotensin system were unchanged and thus unaffected by cardiac unloading. Probably this is due to the therapeutic intention, with which all patients in our study were treated with a LAVD, i.e., the BTT (bridge to transplant) strategy. This means, that by definition cardiac recovery under LVAD therapy must have been incomplete in our patients, since a consecutive heart transplantation was inevitable in all cases. Against this background, it would be important to additionally investigate patients treated with LVADs in a BTR (bridge to recovery) intention, which means that these patients can successfully be weaned from the device due to a sufficient cardiac recovery. But such analyses might prove to be very difficult facing the vanishing low number of BTR patients who account for only 0.3% of all LVADs implanted [5] on the one hand and the highly limited availability of tissue specimen for scientific analyses in those scarce patients on the other hand.

### Contributing factors for LVAD-induced alterations of RAS and β-arrestin pathway components

A considerable number of RAS-/ β-arrestin pathway components was significantly influenced by the underlying etiology of heart failure (i.e., ICM vs. DCM), the extent of left ventricular impairment before LVAD implantation (i.e., the baseline left ventricular ejection fraction), and the duration of LVAD therapy: for example, ACE2 was significantly downregulated after LVAD therapy only in DCM, but not in ICM patients and in those with a shorter than a longer duration of LVAD therapy. This means that several factors exist—beyond cardiac unloading per se—which determine the molecular setup of LVAD-treated hearts, and against this background, it would be most important to investigate, whether this proves true also for clinical effects, i.e., the probability of cardiac recovery.

### Limitations

Our study might have some limitations: Firstly, our patient cohort comprising 20 individuals seems to be rather small. But otherwise this compares well to other studies, which partly investigated even smaller numbers of patients [27, 30, 31], clearly reflecting the overall scarcity of analyzable tissue specimen from these LVAD-treated individuals. Secondly, our analysis is descriptive in nature and, therefore, does not allow causal conclusions regarding the functional relevance of our findings. But clearly that is the domain of interventional studies using highly controllable animal models of the disease, which overcome the essential and insurmountable heterogeneity of patients suffering from end-stage heart failure. Thirdly, we only analyzed patients that got a LVAD device implanted in a BTT (bridge to transplant) intention. This means that by definition no sufficient cardiac recovery took place, since all patients finally had to undergo heart transplantation. Thus, our results rather signify LVAD-induced reverse remodeling on a molecular basis than a real and clinically relevant cardiac regeneration. Lastly, it must be kept in consideration that during LVAD support there might have been unrecorded medication changes in some patients, which further on could have influenced the expression levels of at least few of the proteins under investigation.

## Conclusion

By investigating key components of the RAS and β-arrestin signaling pathways both before and after LVAD implantation, we found complex and remarkably different molecular adaptation patterns, which were additionally influenced by factors such as the etiology of heart failure, the duration of LVAD therapy, or the severity of left ventricular impairment. Future work is necessary to delineate the functional relevance of our findings.
